# Orexin Receptor Targets for Anti-Relapse Medication Development in Drug Addiction

**DOI:** 10.3390/ph4060804

**Published:** 2011-06-14

**Authors:** Luyi Zhou, Wei-Lun Sun, Ronald E. See

**Affiliations:** Department of Neurosciences, Medical University of South Carolina, 173 Ashley Avenue, Charleston, SC 29425, USA

**Keywords:** addiction, hypocretin, orexin, reinstatement, relapse

## Abstract

Drug addiction is a chronic illness characterized by high rates of relapse. Relapse to drug use can be triggered by re-exposure to drug-associated cues, stressful events, or the drug itself after a period of abstinence. Pharmacological intervention to reduce the impact of relapse-instigating factors offers a promising target for addiction treatment. Growing evidence has implicated an important role of the orexin/hypocretin system in drug reward and drug-seeking, including animal models of relapse. Here, we review the evidence for the role of orexins in modulating reward and drug-seeking in animal models of addiction and the potential for orexin receptors as specific targets for anti-relapse medication approaches.

## 1. Background

The orexins (also known as hypocretins) are peptide neurotransmitters expressed in neurons exclusively in the lateral hypothalamus (LH) and the adjacent regions of the perifornical area (PFA) and dorsomedial hypothalamus (DMH) [1-3]. The peptides were discovered virtually simultaneously in 1998 by two groups of researchers - De Lecea *et al.* [1] and Sakurai *et al.* [2]. The former group named the peptides “hypocretins”, because of their hypothalamic location and structural similarity to the incretin family of hormones. The latter group named the peptides “orexins”, due to their appetite-enhancing properties when administered centrally to rats. Orexin occurs in two forms, orexin-A (OXA, or hypocretin 1) and orexin-B (OXB or hypocretin 2), which share 46% homology and are cleaved from a single prepro-orexin precursor polypeptide *via* proteolytic processing [1,2]. Although orexins are found in cell bodies localized exclusively in the hypothalamus, orexin-containing fibers project extensively throughout the brain, including innervations of midbrain structures such as the dorsal raphe and locus coeruleus (LC), and to a lesser extent, the ventral tegmental area (VTA), prefrontal cortex, hippocampus, amygdala, bed nucleus of the stria terminalis, both core and shell regions of the nucleus accumbens (NAc), and within the hypothalamus (arcuate, paraventricular, and ventromedial hypothalamic nuclei) [3-6].

In their original report, Sakurai *et al.* [2] further revealed two G protein-coupled receptors that bind the orexins, OX1R (HCRT1) and OX2R (HCRT2), which share 64% homology. OX1R has 10-fold higher affinity for OXA, whereas OX2R has equal affinity for both peptides [2,7]. OX1R is coupled exclusively to a Gq subclass of G proteins, while OX2R is coupled to both Gq and Gi/o proteins [2]. However, the coupling mechanisms may be different in distinct cell types and have not been fully examined in neurons [8,9]. Similar to the orexin peptides, orexin receptors are widely distributed throughout the brain, including brain regions involved in drug reward and addiction [10-12]. Interestingly, it has also been shown that the orexin receptors show somewhat different patterns of expression. For instance, OX1R is the only orexin receptor expressed in the medial prefrontal cortex [10,12]. However, OX1R levels are fairly low in the LH, while OX2Rs are highly expressed in the LH [11,12]. Orexin receptors are also found to varying degrees in peripheral tissues, including pituitary, adrenal, gonads, kidney, pancreas, heart, and lung [13-15].

Different types of orexin receptor antagonists have been developed over the last few years (for review, see [16]). OX1R antagonists with at least 50-fold selectivity for OX1R over OX2R include SB-334867, SB-674042, SB-410220 and SB-408124. Among these compounds, SB-334867 is the most widely studied drug, due to the favorable preclinical pharmacokinetics of high selectivity and potency. Selective OX2R antagonists include TCS OX2 29, JNJ-10394049, and EMPA, all with at least 250-fold selectivity for OX2R. While none of the selective OX1R or OXR2 antagonists is clinically available, dual orexin receptor antagonists with high affinity for both OX1R and OX2R are in Phase II or III of clinical trials for the treatment of insomnia. These drugs include SB-649868, Almorexant (ACT-078573), and MK-4305 [16].

The widespread orexin innervation and distinctive expression of orexin receptors in the brain suggest that orexin plays multifaceted functions. Initially, orexin was named for appetite-enhancing effects [2]. For example, orexin administration increased feeding behaviors and rewarding properties of food, effects that were reversed by OX1R antagonism [2,17-23]. In addition, opioid-orexin interactions in NAc-hypothalamic projections activate OX1R signaling in the VTA to increase palatable food intake [24]. The orexin system has been shown to be dysfunctional in narcolepsy models, whereby animals exhibit periods of wakefulness interrupted by abrupt brief episodes of sleep. Canine narcoleptic subjects show disruptions in the OX2R gene, which resulted in a mutated orexin receptor [25]. Rodents with a null mutation in the prepro-orexin gene or OX1R and OX2R dual knockout exhibit similar narcoleptic phenotypes [26-28]. Furthermore, infusion of OXA or OXB into the rodent basal forebrain, LC, or lateral ventricles increases wakefulness, while orexin receptor antagonist administration has sleep-promoting effects in both animals and humans (for a review, see [16]). However, only OX2R and OX1R/OX2R, but not OX1R knockout mice display a narcoleptic phenotype, and OX2R and dual orexin receptor antagonists, but not OX1R antagonists decrease wakefulness [16,29]. Together, these findings indicate that the wake-promoting effects of orexins are mainly mediated by OX2R or a combination of OX1R and OX2R, but not OX1R alone. In addition to the importance of the orexin system in arousal and sleep/wake regulation, orexin also plays a critical role in stress responses. For example, orexin knockout mice show attenuated behavioral responses to emotional stress [30]. Administration of OXA and OXB activates the hypothalamic-pituitary-adrenal axis *via* increased adrenocorticotropin hormone and corticosterone release, both of which can be diminished by OX1R or OX2R antagonists [31-35].

Recently, the orexin system has been implicated in substance abuse and addiction. The interaction between the orexin system and drugs of abuse was first suggested by evidence that chronic nicotine treatment increased the expression of orexin and orexin receptors in hypothalamic regions [36,37]. Over the last few years, accumulating studies have confirmed the involvement of orexin signaling *via* OX1R in different stages of drug addiction, supporting the exciting possibility of treating addiction with novel therapeutic interventions that target the orexin system. In this review, we will discuss the role of the orexin system in modulating reward and drug-seeking in different animal models of addiction, and the potential of orexin receptors as targets for anti-relapse medication development.

## 2. Role of Orexin in Drug Addiction and Relapse

Drug addiction is a chronic illness with high rates of relapse (reviewed in [38,39]). It is characterized by compulsive drug-seeking and drug-taking, loss of control in limiting drug intake, and negative emotional states when the drug is no longer available [40]. Relapse to drug use can be triggered by the drug itself, re-exposure to drug-associated cues, and/or stressful events after a period of abstinence. Addicts have a persistent vulnerability to relapse to drug use after months or even years of abstinence, and relapse is considered the most difficult problem in addiction treatment [41]. With the lack of adequate treatment for preventing craving and relapse, drug addiction has enormous social, economic, and medical burdens around the world.

Animal models that approximate the symptoms of human addiction have proven to be a crucial approach to further our understanding of the neurobiological mechanisms of drug addiction. These animal models also offer a means to systematically test new treatment approaches for drug addiction. In the following section, we will focus on three animal models that have been applied to study the role of the orexin system in drug addiction and relapse.

### 2.1. Self-Administration, Extinction, and Reinstatement

Drug self-administration is a widely used animal model across species in preclinical drug addiction research. Of the different animal models, self-administration best mimics conditions of human addiction, whereby the subject controls drug delivery and manifests drug-seeking behaviors. Typically, a catheter is surgically implanted in a jugular vein of the animal. After recovery, animals intravenously self-administer a drug under a standard operant schedule of reinforcement, usually by lever-press or nose poke. Drug infusions are usually paired with stimuli, such as a light and/or tone, which both facilitate the acquisition of self-administration and takes on the properties of a conditioned stimulus. Self-administration is generally divided into an acquisition phase, followed by a maintenance phase, when animals show stable daily responding. A number of variations have been developed in self-administration models across a wide array of abused drugs, including the use of varied schedules of reinforcement, different periods of daily access, and parameters of stimulus presentation.

The most commonly used animal model of relapse following drug self-administration is the extinction-reinstatement paradigm. Once stable self-administration is established and maintained, animals undergo extinction of responding, whereby access to the drug is discontinued. During the extinction phase (which can occur within a single session or across multiple sessions), responding on the device previously resulting in drug delivery and cues has no programmed consequences. After drug-seeking is extinguished to a criterion level, reinstatement of extinguished drug-seeking can be trigged by a priming injection of the drug, a previously drug-paired cue, or a physical (e.g., foot shock) or pharmacological (e.g., yohimbine) stressor. Reinstatement of drug-seeking after extinction implies the restoration of a concrete operant response, and this animal model has relevance to human relapse [42]. A variant of this approach is to maintain an animal in abstinence outside of the drug-taking environment for varied periods of time, and then assess drug-seeking upon immediate return to the environment [43].

#### 2.1.1. Self-Administration

Several studies have examined the role of the orexin system in the acquisition and maintenance of drug self-administration. For cocaine self-administration, OX1R-mediated signaling regulates cocaine intake depending upon the schedule of reinforcement. For example, under a fixed ratio (FR) 1 schedule, acute systemic administration of SB-334867 or intra-VTA administration of OXA did not affect the acquisition and maintenance of cocaine self-administration [44,45]. In addition, chronic SB-334867 treatment (10 mg/kg daily) did not affect the acquisition or maintenance of cocaine self-administration under the same FR1 schedule ([46], Zhou *et al.*, unpublished data). However, when rats were tested under a progressive ratio (PR) schedule, OX1R blockade by systemic SB-334867 (10 mg/kg) significantly reduced the breakpoint of cocaine self-administration, indicating a reduction in motivated drug-seeking [22,47]. Furthermore, intra-VTA SB-334867 administration reduced, while intra-VTA OXA administration increased the breakpoint for cocaine self-administration [45,47]. Since the breakpoint is thought to reflect the animal's motivational limit to gain access to a drug, these data indicate the importance of the orexin system when high levels of motivation are required. The lack of effectiveness of SB-334867 on cocaine self-administration under a FR1 schedule suggests that OX1R-mediated signaling may not be necessary for cocaine-taking under conditions of lower motivation, a point supported by the unpublished observation that SB-334867 dose-dependently decreased cocaine self-administration under a higher demand FR5 schedule [48].

Under an FR schedule, ethanol and nicotine self-administration is decreased by SB-334867 and increased after intracranial OXA administration [49-53]. In a similar manner, systemic injections of SB-334867 significantly reduced breakpoints for ethanol and nicotine self-administration under a PR schedule [50,53]. Thus, as for cocaine, these data suggest that both intake and the motivation to self-administration of ethanol and nicotine are regulated by the orexin system [22,50].

#### 2.1.2. Extinction

On the first day of extinction, animals typically display a burst of activity, with behavioral responses at the same level or higher than those seen during self-administration. The elevated responses can be interpreted as drug-seeking elicited by re-exposure to the drug-paired context, despite the lack of drug availability and drug-paired cues. In rats previously trained for cocaine self-administration, acute pretreatment of SB-334867 on the first day of extinction attenuated the burst response on that day. However, this attenuation effect was only shown on the treatment day, as extinction responding was similar to vehicle-treated animals on subsequent sessions [54]. The sensitivity of SB-334867 treatment also depends on the length of forced abstinence. After one day of abstinence, only a high dose (30 mg/kg) of SB-334867 effectively reduced the context-driven cocaine-seeking. With longer abstinence (14 days) or extinction of cocaine-seeking in a distinct environment that differed from the self-administration environment, even lower doses (10 and 20 mg/kg) of SB-334867 were effective [54]. These changes indicate that the orexin system may be involved in the incubation of drug-seeking after extended periods of abstinence, as the increased cocaine-seeking during abstinence showed greater sensitivity to OX1R antagonism. While no published studies have reported on chronic SB-334867 effects during extinction, our preliminary data show that daily systemic SB-334867 injections prior to each extinction trial facilitate the extinction process and attenuate the cocaine-seeking response throughout the extinction sessions (Zhou *et al.*, unpublished data).

#### 2.1.3. Reinstatement

Following extinction, direct activation of the orexin system can produce reinstatement of a previously extinguished response. Both intracerebroventricular (icv) and intra-VTA administration of OXA reinstate extinguished cocaine-seeking in animals with a prior history of cocaine self-administration [55,56]. SB-334867 administration dose dependently attenuated cue-induced reinstatement, but failed to affect cocaine-primed reinstatement of cocaine-seeking [44]. In addition, treatment with SB-334867 before a single Pavlovian conditioning session, during which cues were passively conditioned with cocaine infusions, did not prevent these cues from reinstating a subsequent extinguished cocaine-seeking response [44]. These results suggest that OX1R-mediated signaling is not necessary during the acquisition of cue-cocaine associations, but rather during the retrieval and expression of cue-paired learning. The fact that the orexin system is involved in cue-induced, but not cocaine-primed reinstatement, indicates that orexin signaling mediates cue-drug associative learning, but not necessarily the primary rewarding effects of the drug itself, as in cocaine-induced reinstatement. Stress-induced reinstatement is also mediated by the orexin system, as SB-334867 dose dependently blocked footshock stress-induced reinstatement of cocaine-seeking [55]. However, this process appears to be independent of VTA signaling, as intra-VTA SB-334867 infusion did not block footshock stress-induced reinstatement [56]. We have also found that systemic SB-334867 attenuated yohimbine stress-induced reinstatement of cocaine-seeking (Zhou *et al.*, unpublished data).

As for other drugs, the reinstatement of alcohol-seeking induced by alcohol-associated cues was attenuated by SB-334867 treatment both immediately after extinction or following protracted abstinence, a model that may better mimic human alcoholism [49,57,58]. In addition, during reinstatement, the alcohol-paired cues increased the immediate early gene product, Fos, in various brain regions, including orexin neurons in the LH, and the degree of alcohol-seeking was positively correlated with Fos expression in the LH [58-60]. SB-334867 also attenuated alcohol-seeking induced by yohimbine stress [49,57]. As for nicotine-seeking, icv infusion of OXA produced reinstatement, an effect blocked by SB-334867 treatment [61]. However, SB-338867 did not block footshock-induced reinstatement [61]. It is possible that stress-induced reinstatement of nicotine-seeking acts through a mechanism independent of OX1R activity. However, a more complete dose response study is required, as only low doses of SB-334867 (5 and 10 mg/kg) were tested for nicotine-seeking, an important point as 30, but not 15 mg/kg of SB-334867 attenuated footshock-induced reinstatement of cocaine-seeking.

### 2.2. Conditioned Place Preference

Conditioned place preference (CPP) is a widely-used animal model for the measurement of the rewarding effects of drugs of abuse. CPP refers to the development of preference for an environment that has previously been paired with noncontingent drug administration (reviewed in [62]). Typically, an animal receives a drug injection and is restricted to one side of a chamber with distinctive visual and tactile cues that differ from the other side of the chamber, which is accessible only after vehicle administration. During training, an association between the environmental cues and the interoceptive properties of the drug is established. On test days, in a drug free condition, access to both sides is unrestricted, and increased time spent on the drug-paired side is an indication of a positive drug rewarding effect. Animals can also undergo a form of extinction-reinstatement with CPP, whereby animals are repeatedly exposed to the chambers in the absence of the drug and then readministered the drug for reinstatement of CPP. Compared to self-administration, CPP is faster and more economical. However, the limited noncontingent drug treatment during the development of CPP does not mimic human drug use. Some discrepant results have been observed between CPP and self-administration studies, suggesting that these two paradigms may reflect different mechanisms of drug reward and addiction [63,64].

In regards to the orexin system, CPP has been primarily used for testing the orexin mediation of morphine CPP, with studies showing a role of the orexin system in the acquisition, expression, and reinstatement of morphine CPP. The acquisition of morphine CPP was associated with increased activity of orexin neurons in the LH, as determined by Fos+/orexin+ double-labeled neurons [65]. Bilateral excitotoxic lesions of the LH blocked the acquisition of morphine CPP. Furthermore, although not separately effective, the combination of unilateral excitotoxic lesions of the LH and intra-VTA administration of SB-334867 on the contralateral side blocked the development of morphine CPP [65]. After re-exposure to drug-associated cues, morphine conditioned rats also exhibited enhanced activation of orexin neurons in the LH, as indicated by increased Fos expression. Importantly, the percentage of Fos positive orexin neurons showed a positive correlation with the amount of time spent in the morphine-paired chamber [66]. Both systemic and intra-VTA administration of SB-334867 reduced the expression of morphine CPP [23,66,67], and reinstatement of morphine CPP *via* administration of rat pancreatic polypeptide in the LH was blocked by systemic SB-334867 [66]. Together, these data indicate the importance of the LH orexin neurons and their projections to the VTA in the development and expression of morphine CPP.

For amphetamine CPP, a high (30 mg/kg), but not low (10 mg/kg) dose of SB-334867 blocked the expression of CPP [46]. Interestingly, and unlike morphine and amphetamine CPP, SB-334867 failed to reduce the expression of cocaine place preference [23]. Similarly, the acquisition or expression of ethanol CPP was not affected by the blockade of OX1R with SB-334867 [68]. These results across drugs of different classes suggest that orexin system mediated rewarding effects may depend upon the unique pharmacological profiles of drugs of abuse, and non-orexin neurotransmitter systems may be sufficient for the acquisition and expression of CPP.

### 2.3. Locomotion

Drug-induced hyperlocomotion and behavioral sensitization are both widely used as measures of the effects of acute and repeated drugs of abuse (see [69-71] for review). Drug-induced hyperlocomotion refers to increased locomotor activity as measured by ambulatory, rearing, and total distance after acute drug administration. Behavioral sensitization refers to a progressive enhancement in locomotor activity following intermittent repeated drug administration at a maintained dose. Animals with initially higher locomotor responding may be more susceptible to developing compulsive drug self-administration [72,73], although disassociations between the two measures have also been reported [74]. In addition, behavioral sensitization may facilitate the acquisition of self-administration or CPP [75-77]. However, while sensitization has been associated with reinstatement of cocaine-seeking, disassociations have been reported for sensitization and the reinstatement of a cocaine CPP [72,78]. While measures of sensitization are useful behavioral assays, the evidence of a relationship between locomotor sensitization and observed human symptoms of addiction remains scarce, thus limiting the validity of this animal model for the study of substance dependence in humans (reviewed in [79]).

To date, studies on the role of the orexin system in drug-induced hyperlocomotion and sensitization are fairly limited. In drug-naïve rats, systemic administration of SB-334867 (10 mg/kg) failed to affect hyperlocomotion induced by acute systemic cocaine administration at the dose of 15 mg/kg [80]. When a lower cocaine dose (10 mg/kg) was used, cocaine-induced hyperlocomotion was significantly blocked by SB-334867 at all three doses of 10, 20, and 30 mg/kg (Zhou *et al.*, unpublished data). However, in rats with a previous cocaine self-administration history, even a high dose of SB-334867 (30 mg/kg) had only a modest effect in reducing cocaine-induced (10 mg/kg) activity as measured by horizontal photobeam beam breaks, and failed to affect total travel distance [44]. For cocaine-induced behavioral sensitization, Borgland *et al.* demonstrated that chronic injection of SB-334867 (10 mg/kg) blocked the development, but not the expression of cocaine sensitization [80].

As for other drugs, ethanol-stimulated hyperactivity was dose-dependently attenuated by SB-334867 pretreatment [68]. Intracranial infusion of SB-334867 failed to affect morphine-induced locomotor hyperactivity and SB-334867 (20 mg/kg) also did not affect the development or expression of behavioral sensitization [23,81]. However, acute SB-334867 (30 mg/kg) reduced the expression of amphetamine sensitization after a period of abstinence from drug taking [82]. Furthermore, it has been demonstrated that SB-334867 reduced dopamine outflow in the NAc shell evoked by acute amphetamine treatment [82] and that activation of orexin neurons in hypothalamic regions was increased during the expression of amphetamine sensitization [83].

## 3. Additional Considerations

### 3.1. Role of Orexin 2 Receptor Signaling in Drug Addiction

Most studies on the role of orexin in drug addiction have focused on the role of OXA and the OX1R. The role of OX2R in addiction has not been well explored, due to the lack (until recently) of selective OX2R antagonists. *In vitro* studies indicate that OX2R-mediated signaling may also be involved in drug addiction. For example, OXB mediates VTA synaptic plasticity by activating OX2R [84]. In addition, OXA stimulated VTA dopamine neuron firing was only partially blocked by OX1R antagonists (SB-674042 and SB-408124), but fully blocked by the OX2R antagonist, EMPA [85]. In a study on cocaine self-administration in rats, the putative orexin-2 receptor antagonist, 4-PT, failed to affect cocaine intake or reinstatement to cocaine-seeking [44]. However, a recent study indicated that JNJ-10397049, another specific OX2R antagonist, dose-dependently reduced ethanol self-administration, and attenuated the acquisition, expression, and reinstatement of ethanol CPP and ethanol-induced hyperactivity in mice [86]. These results implicate an involvement of OX2R mediated signaling, at least for ethanol addiction. Future exploration of the role of OX2R in addiction with other drugs is warranted.

### 3.2. Sex Differences in Orexin System Mediation of Addiction

Gender differences in addition have been previously documented, with a number of studies suggesting that women are more susceptible to the addictive properties of drugs of abuse as compared to men (see [87] for a review). For example, women start using cocaine at an earlier age with more severe usage than men and develop cocaine dependency at a faster rate. Preclinical studies have provided increasing evidence that females differ from males at different stages of the addiction process. For example, female rats acquire self-administration of cocaine, heroin, methamphetamine, and nicotine faster than males [88-92]. Compared to males, females also exhibit greater resistance to extinction, and reinstate more to cocaine- or yohimbine stress-induced cocaine-seeking [93-96] However, under some conditions, male rats show higher reinstatement to cocaine-seeking induced by cocaine-conditioned cues than females [97]. In the CPP model, compared to male rats, females require fewer sessions and lower doses to acquire CPP induced by cocaine and morphine [98,99]. In addition, females are more sensitive to the locomotor stimulating effects of cocaine than males [100].

Although the exact mechanisms underlying these sex differences remains unclear, it has been demonstrated that gonadal hormones play a critical role. For example, estrogen facilitates drug-seeking, drug-induced CPP, and locomotor sensitization, while progesterone counteracts the effects of estrogen [101-106]. Interestingly, sex and estrous cycle differences in the orexin system have also been reported. Female rats express higher levels of OXA, prepro-orexin, and OX1R in the hypothalamus as compared to male rats [14,107,108]. Within the same brain area, the expression of PPO, OX1R, and OX2R increases selectively in female rats during proestrus, with no changes observed in estrus, diestrus, or in males (reviewed in [109]). These findings provide a biological basis for potential sex and estrous cycle differences that may occur in the orexin system due to drugs of abuse. While no published studies have examined this issue, our preliminary data show that OX1R blockade by SB-334867 failed to alter cue-induced reinstatement of cocaine-seeking in females. The sex differences in OX1R regulation of cue-induced cocaine-seeking may be due to sexually dimorphic regulation of orexin target regions in mesocorticolimbic circuits. Further studies on sex differences in the orexin system will improve potential gender-specific approaches to addiction treatment.

## 4. Conclusions

In conclusion, growing evidence has confirmed the critical role of the orexin system in drug addiction. Specifically, OX1R-mediated signaling is involved in drug-taking and drug-seeking behavior, including self-administration, extinction, and relapse. Other measures of the behavioral effects of drugs of abuse, including CPP and behavioral sensitization, confirm a contributing role for orexin. Specific brain regions likely mediate the action of orexin in these behaviors, as previous studies have indicated that distinct neural pathways mediate motivated drug-seeking [43,110]. As proposed by Harris and Aston-Jones [111], distinct subpopulations of orexin neurons in the hypothalamus may play different roles. LH orexin neurons seem to mediate reward- and cue-related processing *via* VTA activation, whereas DMH and PFA orexin neurons appear to mediate stress-related actions through regulation of the NAc, paraventricular nucleus of hypothalamus, and central nucleus of amygdala. Thus, the inhibitory effect of OX1R-blockade on drug-seeking behavior across animal models and under different conditions may result from actions on neurocircuitry that influence drug-seeking in a modality specific manner (e.g., cues vs. stress).

Orexin likely influences addiction-related behaviors *via* interactions with norepinephrine (NE) and corticotropin-releasing factor (CRF) systems [112]. NE and CRF systems play an important role in several aspects of addiction, including stress-induced relapse and increased drug intake and drug-seeking following prolonged exposure and abstinence [113]. Footshock stress is CRF dependent and activates orexin neurons in the PFA and DMH [66]. On the other hand, OXA infusion increases the release of corticosterone and NE [114,115]. Both NE antagonists (ip) and CRF antagonists (icv) block reinstatement of cocaine-seeking induced by icv OXA infusion, indicating an interplay of these systems with the actions of orexin [55]. However, some evidence suggests that CRF and orexin systems may be independent, at least within the VTA, as CRF antagonism within the VTA failed to attenuate cocaine-seeking induced by intra-VTA OXA perfusion, while intra-VTA administration of an OX1R antagonist did not block footshock stress-induced reinstatement [56]. Further clarification of the mechanisms for these interactive neurotransmitters will help us to understand the multiple roles of the orexin system in mediating drug-seeking.

OX1R is coupled exclusively to a Gq subclass of G proteins, while OX2R is coupled to both Gq and Gi/o proteins [2]. However, the underlying intracellular mechanism still remains understudied, with only a few published *in vitro* experiments. The application of OXA potentiated N-methy-D-aspartate receptor (NMDAR) currents in VTA dopamine neurons, which was blocked by the application of SB-334867 or PLC/PKC inhibitors, indicating that synaptic insertion of NMDAR induced by OXA is OX1R and PLC/PKC dependent [80]. However, in an OX1R- or OX2R-independent manner, OXB also potentiated NMDAR-mediated transmission *via* a PKC-mediated pathway [84]. A study using H295R cells indicated the involvement of mitogen-activated protein kinase pathway in response to *in vitro* application of orexin. Both extracellular receptor kinase 1/2 and p38 were rapidly and transiently activated in response to both OXA and OXB [116]. Taken together, these results demonstrate that although coupling to different types of G protein, OXA and OXB might share similar intracellular pathways. However, it must be reiterated that orexin receptor antagonists are somewhat non-selective. For example, the most widely used OX1R antagonist, SB-334867, has 50-fold selectivity for OX1R to OX2R. Thus, it is possible that the application of an OX1R antagonist may also mask the effect of OX2R activity. Elucidation of the orexin signaling pathways and their interaction with other pathways *in vivo* is necessary to further understand the role of orexin in drug addiction and develop anti-relapse medication targeting orexin receptors, especially OX1R. To improve targeting and reduce potential side effects, development of more selective OX1R antagonists is especially warranted.

Finally, our preliminary data demonstrate a sexually dimorphic effect of OX1R inhibition on drug-seeking behavior. However, the neuronal mechanisms behind the sexual dimorphism remain unknown. Future experiments will need to characterize the expression and localization of orexin receptors in different brain areas, and the pharmacokinetics of orexin peptides and antagonists in both sexes. The influence of gonadal hormones on the orexin system is also likely to be a major contributor. Understanding the differential role of the orexin system in drug addiction will help facilitate gender-specific treatment approaches.
